# Supplementary material for: Numbers and Mortality Risk of Hypertensive Patients with or without Elevated Body Mass Index in China
Source: Int J Environ Res Public Health. 2021 Dec 23;19(1):116. doi: 10.3390/ijerph19010116 (PMC8750230; doi:10.3390/ijerph19010116)
Supplement: Supplementary file 1 [file ijerph-19-00116-s001.zip › ijerph-1502799-supplementary.pdf]

Supplementary Table S1 Hazard ratios of premature death according to BMI and blood pressure categories in the different age and sex subgroup[1,2]

	Model 1*		Model 2†		Model 3‡	
	HR	95%CI	HR	95%CI	HR	95%CI
**Age<65**						
**All-cause mortality**						
Normal BP and BMI	Reference		Reference		Reference	
Normal BP but elevated BMI	0.44	0.14-1.42	0.48	0.11-2.04	0.61	0.14-2.72
Hypertension but normal BMI	1.19	0.80-1.76	1.18	0.75-1.85	1.00	0.63-1.60
Grade 1 Hypertension and elevated BMI	1.20	0.71-2.04	1.33	0.72-2.46	1.51	0.74-3.09
Grade 2 Hypertension and elevated BMI	0.79	0.37-1.66	0.77	0.34-1.73	0.76	0.33-1.76
Grade 3 Hypertension and elevated BMI	1.93	1.06-3.50	2.09	1.15-3.80	2.27	1.20-4.29
**Premature death**						
Normal BP and BMI	Reference		Reference		Reference	
Normal BP but elevated BMI	0.59	0.29-1.20	0.65	0.29-1.47	1.79	0.67-4.80
Hypertension but normal BMI	0.97	0.65-1.46	1.33	0.86-2.06	1.40	0.90-2.17
Grade 1 Hypertension and elevated BMI	0.76	0.47-1.24	0.81	0.40-1.66	2.28	0.91-5.72
Grade 2 Hypertension and elevated BMI	0.27	0.04-1.90	0.80	0.28-2.24	2.31	0.68-7.78
Grade 3 Hypertension and elevated BMI	1.96	0.73-5.28	1.40	0.50-3.89	3.98	1.14-13.82
**Age≥65**						
**All-cause mortality**						
Normal BP and BMI	Reference		Reference		Reference	
Normal BP but elevated BMI	0.88	0.74-1.05	0.97	0.78-1.20	1.55	1.16-2.07
Hypertension but normal BMI	3.34	2.90-3.85	1.81	1.54-2.14	1.92	1.62-2.27
Grade 1 Hypertension and elevated BMI	1.59	1.24-2.03	0.97	0.73-1.29	1.60	1.12-2.28
Grade 2 Hypertension and elevated BMI	2.76	1.90-3.40	1.46	0.98-2.17	2.64	1.65-4.23
Grade 3 Hypertension and elevated BMI	4.85	3.23-7.29	2.89	1.89-4.40	4.94	3.07-7.95
**Premature death**						
Normal BP and BMI	Reference		Reference		Reference	
Normal BP but elevated BMI	0.89	0.74-1.08	0.96	0.76-1.21	1.68	1.22-2.31
Hypertension but normal BMI	3.08	2.64-3.60	1.86	1.55-2.25	1.99	1.65-2.41
Grade 1 Hypertension and elevated BMI	1.47	1.12-1.92	0.98	0.72-1.33	1.76	1.19-2.60
Grade 2 Hypertension and elevated BMI	2.28	1.49-3.49	1.30	0.82-2.07	2.62	1.53-4.48
Grade 3 Hypertension and elevated BMI	4.85	3.17-7.42	3.10	1.99-4.84	5.89	3.55-9.79

**Male**						
**All-cause mortality**						
Normal BP and BMI	Reference		Reference		Reference	
Normal BP but elevated BMI	1.10	0.71-1.72	1.13	0.72-1.77	1.34	0.82-2.20
Hypertension but normal BMI	2.69	2.41-3.00	1.34	1.19-1.52	1.34	1.18-1.52
Grade 1 Hypertension and elevated BMI	1.78	1.15-2.75	1.31	0.82-2.08	1.50	0.91-2.47
Grade 2 Hypertension and elevated BMI	1.45	0.69-3.06	0.73	0.33-1.63	0.75	0.32-1.73
Grade 3 Hypertension and elevated BMI	4.67	2.22-9.85	2.79	1.32-5.90	3.34	1.54-7.23
**Premature death**						
Normal BP and BMI	Reference		Reference		Reference	
Normal BP but elevated BMI	0.87	0.67-1.13	0.99	0.73-1.35	1.56	1.03-2.35
Hypertension but normal BMI	2.48	2.05-2.99	1.75	1.39-2.19	1.82	1.44-2.29
Grade 1 Hypertension and elevated BMI	1.19	0.84-1.69	0.88	0.59-1.33	1.37	0.82-2.28
Grade 2 Hypertension and elevated BMI	1.87	1.11-3.13	1.34	0.76-2.36	2.24	1.17-4.29
Grade 3 Hypertension and elevated BMI	4.61	2.71-7.87	3.30	1.87-5.80	5.02	2.65-9.52
**Female**						
**All-cause mortality**						
Normal BP and BMI	Reference		Reference		Reference	
Normal BP but elevated BMI	0.90	0.72-1.14	0.87	0.66-1.14	1.65	1.13-2.41
Hypertension but normal BMI	4.36	3.59-5.30	1.62	1.30-2.03	1.72	1.37-2.16
Grade 1 Hypertension and elevated BMI	1.78	1.29-2.47	0.87	0.60-1.26	1.76	1.11-2.79
Grade 2 Hypertension and elevated BMI	4.01	2.58-6.23	1.45	0.90-2.35	3.40	1.89-6.14
Grade 3 Hypertension and elevated BMI	5.39	3.31-8.77	1.94	1.16-3.23	4.50	2.46-8.25
**Premature death**						
Normal BP and BMI	Reference		Reference		Reference	
Normal BP but elevated BMI	0.93	0.72-1.20	0.87	0.63-1.20	1.91	1.23-2.96
Hypertension but normal BMI	3.71	2.95-4.67	1.83	1.39-2.40	1.96	1.48-2.58
Grade 1 Hypertension and elevated BMI	1.91	1.35-2.69	1.15	0.78-1.70	2.69	1.62-4.47
Grade 2 Hypertension and elevated BMI	2.66	1.49-4.74	1.16	0.61-2.21	3.27	1.54-6.94
Grade 3 Hypertension and elevated BMI	4.87	2.79-8.50	2.27	1.26-4.09	6.26	3.11-12.62

China Health and Nutrition Survey (CHNS) cohort data with baseline and last time been followed up data was used to fit the cox model.

[1]Elevated BMI: body mass index ≥ 24.0.

[2]Hypertension: categories of blood pressure: normal (SBP < 120 mm Hg and DBP < 80 mm Hg), high normal (SBP 120-139 mm Hg and/or DBP 80-89 mm Hg), and hypertensive (SBP

≥ 140 mmHg and/or DBP ≥ 90 mmHg) for adults over 18 years. Hypertension is further divided into three grades, grade 1 (SBP 140-159 mm Hg and/or DBP 90-99 mm Hg), grade 2 (SBP 160-179 mm Hg and/or DBP 100-109 mm Hg), and grade 3 (SBP ≥ 180 mm Hg and/or DBP ≥ 110 mm Hg).

*Model 1: adjusted for age, sex.

†Model 2: adjusted for age, sex, educational level, marital status, whether rural residents, history of drinking or smoking.

‡Model 3: adjusted for age, sex, educational level, marital status, whether rural residents, history of drinking or smoking, body mass index, previous history of diabetes mellitus, CVD, or cancer.
